# Long-term oxygen therapy to reduce length of hospital stay in COVID-19

**DOI:** 10.1590/1806-9282.20231379

**Published:** 2024-07-19

**Authors:** Douglas Inomata Cardoso da Silva, Letícia Yumi Ishimoto, Estefânia Aparecida Thomé Franco, Maércio Souza Cícero dos Santos, Luís Fernando Pereira Brizola, Camila Aparecida Colombo, Edris Guardiano Savadkouhi, Luiz Henrique Soares Machado, Suzana Erico Tanni, Robson Prudente

**Affiliations:** 1 São Paulo State University (UNESP), Medical School, Distrito de Rubião Junior s/n – Botucatu (SP), Brazil.

**Keywords:** COVID-19, SARS-CoV-2, Hospitalization, Hypoxemia, Oxygen inhalation therapy

## Abstract

**OBJECTIVE:**

The aim of this study was to evaluate the efficacy of long-term oxygen therapy as a strategy to reduce hospitalization time in patients affected by COVID-19.

**METHODS:**

Between April and December 2021, COVID-19 patients with stable clinical conditions needing supplementary oxygen therapy during hospitalization were oriented to have hospital discharge with long-term oxygen therapy and reassessment after 15 days.

**RESULTS:**

A total of 62 patients were evaluated and, 15 days after discharge, 69% of patients had suspended long-term oxygen therapy, with no difference between the groups admitted to the intensive care unit or the ward (p=0.319). Among the individuals who needed to maintain long-term oxygen therapy, in addition to worse P/F ratio (265±57 vs. 345±51; p<0.001) and lower partial pressure of oxygen (55±12 vs. 72±11 mmHg; p<0.001), were those more obese (37±8 vs. 30±6 kg/m2; p=0.032), needed more time for invasive mechanical ventilation (46±27 vs. 20±16 days; p=0.029), had greater persistence of symptoms (p<0.001), and shorter time between the onset of symptoms and the need for hospitalization (7 [2–9] vs. 10 [6–12] days; p=0.039).

**CONCLUSION:**

Long-term oxygen therapy is an effective strategy for reducing hospitalization time in COVID-19 patients, regardless of gravity. Additionally, more obese patients with persistence of respiratory symptoms, faster disease evolution, and more days of invasive mechanical ventilation needed to maintain the long-term oxygen therapy longer.

## INTRODUCTION

Despite its high mortality rate, most individuals affected by COVID-19 overcome the acute phase and recover. However, for those who survive, the disease can be associated with varying degrees of functional impairment. One of the challenges faced is the care of these patients after the acute phase, especially those subjected to more invasive treatments^1,2^.

A study conducted in Italy in 2020 identified that, among patients who had recovered from the acute phase of the disease, 87.4% reported the persistence of at least one symptom, the most prevalent being fatigue, followed by shortness of breath^3^. These were also the two main complaints identified in another study conducted in the United Kingdom in the same year^4^. Several factors are associated with the persistence of these manifestations, such as persistent chronic inflammation, organic changes related to the disease, prolonged hospitalization, and associated intensive care^3,5^.

In this sense, fatigue and shortness of breath are the two main symptoms that lead to post-COVID-19 functional impairment, and therapeutic and pulmonary rehabilitation measures should be started early, with the aim of mitigating the consequences of the disease. Due to the persistence of refractory hypoxemia, which is considered an important severity factor, oxygen therapy has become one of the mainstays of treatment and can be instituted at home. Thus, before hospital discharge, the need and possibility of indicating it to the patient must be evaluated^6^.

In addition to ensuring tissue oxygenation in patients with some degree of pulmonary impairment, long-term oxygen therapy (LTOT) is a strategy that allows patients who are already recovering and in the final phase of treatment to be discharged from the hospital, which reduces the length of hospital stay (and its associated complications) and makes room for patients with more serious conditions. Not all patients have an indication for the use of home oxygen; therefore, it is necessary to investigate and understand the characteristics and clinical conditions associated with those who need it after hospital discharge.

Therefore, the authors designed the present study to evaluate the effectiveness of prolonged home oxygen therapy as a strategy to reduce hospitalization time in patients hospitalized for COVID-19 as well as the clinical characteristics of these patients after hospital discharge.

## METHODS

This study included patients hospitalized at the Clinical Hospital of Botucatu Medical School (HCFMB) between 2020 and 2021, affected moderately or severely by COVID-19 who met the criteria for indication of home oxygen therapy (stable for over 48 h with SpO_2_ ≤ 92% on room air, oxygen flow ≤ 3 L/min, and either PaO_2_ ≤ 60 mmHg or SaO_2_ ≤ 92% in arterial blood). They were evaluated after 15 days of home oxygen therapy.

After approval by the Research Ethics Committee of the Botucatu Medical School (approval number: 4999534) and acceptance of the informed consent form, each patient was analyzed and classified, taking into account sociodemographic data and clinical characteristics during hospitalization such as respiratory symptoms and presence of comorbidities, in addition to the drugs used.

Patients over 18 years of age, of both sexes, diagnosed with COVID-19 and admitted to the HCFMB, who had an indication for home oxygen therapy after hospital discharge were included in the study. Patients who died before reassessment, were lost to follow-up, or did not sign the informed consent form were excluded from the study.

Aspects related to hospitalization, such as pulmonary impairment shown on tomography, maximum oxygen flow during hospitalization, need for intensive care, dependence on invasive ventilatory support, and renal replacement therapy, were also evaluated. The period, in days, from the onset of symptoms to the need for hospitalization, total length of hospital stay, time in the intensive care unit (ICU), and use of invasive mechanical ventilation (IMV) were also measured if intensive care was required.

Additionally, the possibility of suspending LTOT was evaluated, investigating how many patients needed to remain on oxygen therapy and why as well as checking the parameters such as body composition, handgrip strength, arterial blood gas, blood pressure, and the 4-min step test that were taken into account in this decision.

Descriptive statistics were used to describe the characteristics of all participants. Variables with normal distribution were expressed as mean values, standard deviations, medians, and 25–75% percentiles for nonparametric variables. Student's t-test compared normally distributed variables, while the Mann-Whitney test assessed non-normally distributed ones. The chi-square test examined binary qualitative variables with frequencies >5, and the McNemar test compared proportions within the same group. Relevant correlations were investigated using Pearson's and Spearman's correlation. A 5% significance level was used for all tests. The Jamovi version 2.3 statistical package (The Jamovi project, Sydney, Australia) was used.

## RESULTS

Between April and December 2021, 62 COVID-19 patients who had an indication for the use of LTOT after hospitalization were evaluated. Of these, 51% (n=32) required intensive care, with a median length of stay of 11 (6–33.5) days, and of these, 50% (n=16) required invasive ventilatory support for approximately 21.5 (9.5–32) days and 19% (n=6) had an indication for acute renal support. Bacterial co-infection requiring antibiotic therapy throughout hospitalization was present in approximately 79% (n=49) of the patients.

The general characteristics of the patients included in the sample according to the severity of COVID-19 (whether or not intensive care is needed) are shown in Table 1, and impairment on chest CT is presented in Table 2.

**Table 1:** Characteristics of individuals according to the severity of COVID-19.

Variables	ICU (n=32)	Ward (n=30)	p
Age (years)	56±13.5	68±15	0.001^a^
Sex, F/M	16/16	14/16	0.793^b^
BMI, kg/m²	33±8.5	30±4.6	0.267^a^
Smoking, yes/no	16/16	17/13	0.599^b^
Smoking history, pack years	0 (0–41.5)	9 (0–31.5)w	0.438^c^
Evolution of symptoms, days^1^	8.5 (6.75–11.3)	7 (4–11)	0.533^c^
Length of hospital stay, days	25.5 (15–43)	15 (12–24)	0.001^c^
Persistence of symptoms^2^, yes/no	12/20	13/17	0.640^c^
PaO_2_ (admission), mmHg	63 (55–69)	65 (58–74)	0.187^c^
PaO_2_ (reevaluation), mmHg	69±13	66±14	0.404^a^
PaO_2_/FiO_2_ (admission)	110 (79–219)	220 (185–255)	0.002^c^
PaO_2_/FiO_2_ (reevaluation)	327±64	313±68	0.402^a^
CT ≥50%, yes/no	24/8	14/15	0.031^b^
Previous lung disease, yes/no	6/26	7/23	0.658^b^
Multimorbidity, yes/no	23/9	23/7	0.667^b^

Values expressed as mean ± standard deviation or median (25–75%). PaO_2_: partial pressure of oxygen; FiO_2_: fraction of inspired oxygen; CT: computed tomography (chest).

1 Time between symptoms onset and hospital admission.

2 Symptoms of dyspnea and fatigue after hospital discharge.

a Student's t-test;

b chi-square or Fisher's exact test;

c Mann-Whitney U test.

**Table 2:** Previous comorbidities according to the pulmonary injury.

Previous comorbidities	CT <50% (n=23)	CT ≥50% (n=38)	p
Cardiovascular, yes/no	17/6	26/12	0.649
Endocrinology, yes/no	8/15	15/23	0.714
Kidney, yes/no	3/20	3/35	0.513
Neurological, yes/no	4/19	4/34	0.461
Lung, yes/no	7/16	6/32	0.208
mental, yes/no	2/21	1/37	0.551
Multimorbidity, yes/no	19/4	26/12	0.222

Chi-square or Fisher's exact test.

After 15 days of hospital discharge, approximately 31% (n=19) met the criteria for continuing therapeutic oxygen use. Among these individuals, there was no distinction in maintaining LTOT between those who required critical care or exhibited lung involvement on chest CT scans. However, in addition to poorer P/F ratio and lower partial pressure of oxygen, those who needed to continue LTOT were more obese, required a longer duration of invasive mechanical ventilation, experienced greater persistence of symptoms, and had a shorter interval between symptom onset and the need for hospitalization (Table 3).

**Table 3:** Characteristics of the individuals according to the need to maintain the long-term oxygen therapy.

Characteristics	No LTOT (n=43)	LTOT (n=19)	p
Age, years	60±15	64±15	0.365^a^
Sex (M/F), n	22/22	10/9	0.934^b^
BMI, kg/m²	30±6	37±8	0.032^a^
Smoking, yes/no	18/26	11/8	0.334^b^
Smoking history, pack years	0 (0–35)	15 (0–30)	0.813^c^
Evolution of symptoms, days^1^	10 (6–12)	7 (2–9)	0.039^c^
Persistence of symptoms^2^, yes/no	11/32	14/5	<0.001^b^
PaO_2_ (admission), mmHg	64 (58–71)	63 (56–71)	0.561^c^
PaO_2_ (reevaluation), mmHg	72±11	56±12	<0.001^a^
PaO_2_/FiO_2_ (admission)	207 (136–261)	185 (84–228)	0.089^c^
PaO_2_/FiO_2_ (reevaluation)	345±51	265±57	<0.001^a^
CT ≥50%, yes/no	27/15	11/8	0.633^c^
Previous lung disease, yes/no	9/34	4/15	1.000^c^
Multimorbidity, yes/no	29/14	17/2	0.114^b^
Length of hospital stay, days	21 (14–30)	21 (12–36)	0.945^c^
Length of ICU stay, days	11 (6–30)	14 (6–65)	0.498^c^
IMV time, days	20±16	46.5±27	0.029^a^

Values expressed as mean ± standard deviation or median (25–75%). PaO_2_: partial pressure of oxygen; FiO_2_: fraction of inspired oxygen; ICU: intensive care unit; IMV: invasive mechanical ventilation.

1 Time between symptoms onset and hospital admission.

2 Symptoms of dyspnea and fatigue after hospital discharge.

a Student's t-test;

b chi-square or Fisher's exact test;

c Mann-Whitney U test.

Furthermore, of the total number of patients, seven needed to maintain LTOT for more than 3 months, and, among them, it was observed that cardiovascular disorders patients showed a greater need for the maintenance of LTOT (p=0.006).

No significant correlations were found among the other studied variables.

## DISCUSSION

While it was anticipated that ICU individuals with COVID-19 would display more severe symptoms and prolonged hospitalizations, it is important to note that these patients were younger and showed no disparities in comorbidities or previous multimorbidities. Typically, advanced age is linked with higher mortality rates and a greater need for critical care. Our sample, however, comprised of younger patients, prompting further investigation into unanalyzed factors like inflammatory markers or specific laboratory parameters that may account for these differences^4^.

Unlike chronic pulmonary conditions, which have well-established criteria and protocols in the literature on the indications and benefits of LTOT, the use of oxygen after hospital discharge does not have specific guidelines defined for patients with sequelae of COVID-19^7^. In contrast, a study by Weerahandi et al.^8^. demonstrated that about 13.5% of approximately 160 COVID-19 patients who needed home oxygen continued LTOT 30–40 days after discharge. Additionally, Loerinc et al.'s study with 310 COVID-19 patients found that 13% required LTOT, but post-discharge duration was unspecified^9^.

COVID-19 itself can lead to chronic hypoxemia, resulting from damage to both the parenchyma and pulmonary vasculature. However, the precise mechanism of chronic hypoxia requires further exploration^10^. In our sample, pulmonary impairment on chest CT during hospitalization was not associated with the need for LTOT. Nevertheless, patients requiring oxygen for > 15 days exhibited poorer oxygenation, experienced ongoing symptoms of dyspnea and fatigue, had higher obesity rates, longer mechanical ventilation periods, and a quicker progression from symptom onset to hospital admission.

Obese patients, especially those with obesity hypoventilation, generally benefit from oxygen therapy when noninvasive ventilation (NIV) alone cannot correct hypoxemia, and in such cases, it can be used in conjunction with oxygen^11^. An observational study by Priou et al.^12^ found that supplemental oxygen therapy was an independent risk factor for mortality in 130 patients with obesity hypoventilation. However, there is no consensus on its benefits, particularly due to potential adverse effects like exacerbating respiratory acidosis or inducing compensatory metabolic alkalosis^13,14^.

We hypothesized that hypoxemia might be attributed to obesity hypoventilation since chest tomography did not indicate significant impairment in the group requiring ongoing oxygen therapy. This could potentially eliminate pulmonary sequelae of COVID-19. However, confirming this hypothesis requires additional post-hospital discharge chest CT analysis and possibly pulmonary function testing, including diffusing capacity for carbon monoxide^15^.

Furthermore, extended periods of mechanical ventilation in these patients likely contributed to increased muscle weakness, potentially leading to hypoventilation and hypoxemia, particularly in those who were more obese. Prolonged mechanical ventilation is known to result in respiratory muscle weakness. In obese individuals, alterations in respiratory mechanics ultimately lead to reduced ventilation in the lower lobes, affecting the ventilation–perfusion ratio and causing hypoxemia^16^.

Patients requiring LTOT had a shorter time from symptom onset to hospitalization, and our findings align with literature reporting similar timelines for hospitalization and ICU admission in Brazil's initial COVID-19 cases. This underscores rapid clinical deterioration. While symptom onset is a valuable baseline for prognostic models, studies on worse outcomes due to swift disease progression remain limited^17^.

The high rate of individuals who have persistent impairment after acute infection by COVID-19 has impacted both the quality of life and the health systems of those affected. The persistence of symptoms for weeks or months after the initial manifestation of the disease is called long COVID or post-COVID syndrome, and the most frequent symptoms are related to the presence of fatigue, dyspnea, cough, muscle pain, arthralgia, palpitations, chest pain, anosmia, dysgeusia, brain clouding, insomnia, anxiety, and depression^18,19^.

The exact mechanism behind the long COVID remains unclear due to a lack of understanding about sustaining factors. However, indications suggest persistent symptoms could be linked to acute phase organic damage, its extent, and system recovery time. Other factors may include ongoing inflammation, immune response, autoantibody generation, virus persistence, nonspecific effects of hospitalization, post-ICU syndrome, SARS-CoV-2-related complications, comorbidities, and acute-phase drug side effects. Additionally, physical deconditioning, psychological issues, post-traumatic stress, and social/financial impacts may contribute to prolonged symptom persistence^18-23^.

Despite differences in our sample, it is crucial to note that patients requiring LTOT after 15 days post-hospital discharge did not necessarily have a greater COVID-19 severity during hospitalization. In other words, there was no significant difference in terms of worse tomographic compromise or critical care necessitating LTOT maintenance. This underscores that discharging individuals who only require oxygen therapy, irrespective of initial severity, and reevaluating the need for LTOT after 15 days, is an effective therapeutic and administrative strategy. This approach not only frees up hospital beds but also led to 69% of the sample being discharged from LTOT after reassessment, highlighting its dual benefits.

Finally, our study has some limitations. Data that could explain findings, like the influence of initial lab parameters on critical care need, were not analyzed. Moreover, post-LTOT discharge reassessment with chest CT and pulmonary function tests could clarify hypoxemia due to hypoventilation in those requiring extended oxygen therapy. These unanswered questions serve as a stimulus for further research in this population.

## CONCLUSION

The indication of LTOT for patients affected by severe COVID-19 has proven to be an effective strategy for reducing the hospitalization time of patients with persistent hypoxemia. In addition, factors related to worse P/F ratio, length of stay, and greater lung impairment may influence the severity of the disease; and aspects that go beyond poor oxygenation such as maintenance of respiratory symptoms, obesity, duration of mechanical ventilation, and shorter time between the onset of symptoms and hospital admission may influence the need to maintain LTOT.
